# Non-bacterial Thrombotic Endocarditis (NBTE) in the Absence of Malignancy or Lupus Anticoagulant/Antiphospholipid Antibodies: A Case Report

**DOI:** 10.7759/cureus.59336

**Published:** 2024-04-30

**Authors:** Patryk Patrzałek, Tomasz Zawada, Łukasz Stolarski, Magdalena Kamińska, Wiesław Kaczmarek

**Affiliations:** 1 Surgery, Rawicz District Hospital, Rawicz, POL; 2 Intensive Care Unit, Rawicz District Hospital, Rawicz, POL

**Keywords:** covid-19, renal replacement therapy (rrt), medical intensive care unit (micu), severe ards, acute respiratory distress syndrome [ards], non-bacterial thrombotic endocarditis

## Abstract

Non-bacterial thrombotic endocarditis (NBTE) is a very rare condition characterized by sterile thrombi formation on cardiac valves and is often associated with hypercoagulation states, such as malignancy and autoimmune disorders.

We present the case of a 74-year-old patient admitted to the intensive care unit with acute respiratory failure, who had a history of COVID-19 infection five months prior to admission, despite having received certified vaccination. The patient developed NBTE involving the mitral valve, alongside acute respiratory distress syndrome (ARDS). In spite of the exclusion of cancer and systemic connective tissue disorders, the patient's condition rapidly deteriorated, leading to treatment-resistant multi-organ failure and demise, despite aggressive management, including anticoagulation therapy, mechanical ventilation, and renal replacement therapy.

This case underscores the need for further research into the mechanisms underlying NBTE in the absence of traditional risk factors. Additionally, it highlights the importance of long-term anticoagulant therapy in NBTE management to mitigate the risk of embolic complications. Our case contributes to the growing body of literature identifying a subset of NBTE cancer-free patients with distinct characteristics, including those associated with current or past COVID-19 infection.

## Introduction

Non-bacterial thrombotic endocarditis (NBTE), a rare condition characterized by sterile thrombi formation on healthy or mildly degenerated cardiac valves, was first described by Ziegler in 1888 [1]. NBTE is often associated with hypercoagulation states, such as malignancy and autoimmune disorders [2]. The primary cause of morbidity in NBTE is attributed to embolic complications, which primarily affect the brain.

NBTE presents with a heightened prevalence among females and is commonly associated with advanced-stage cancers. Notably, lung cancer stands out as the predominant cause of cancer-associated NBTE (Ca-NBTE) [3-5]. Recent studies have indicated a significant association between COVID-19 and infection-induced hypercoagulation persisting up to 180 days after the initial infection [6-8].

In this case study, we outline the progression of illness in a 74-year-old individual admitted to the intensive care unit due to acute respiratory failure. Despite being fully vaccinated against COVID-19, the patient had previously contracted the virus five months before admission. Throughout the hospitalization, the patient had been diagnosed with NBTE, impacting the mitral valve. Alongside NBTE, acute respiratory distress syndrome (ARDS) had been diagnosed.

## Case presentation

A 74-year-old patient of Caucasian ethnicity with a BMI of 43.2 was admitted to the intensive care unit with features indicative of severe respiratory insufficiency (PaO_2_/FiO_2_ 96, SatHb 75%). In gasometry, the result confirmed partially compensated respiratory acidosis. Cultures did not show bacterial growth. Inflammatory parameters were as presented in the table below (Table 1). Hemodynamically, there were no signs of circulatory failure. In the period leading up to admission, the patient had been managed in primary care due to an upper respiratory tract infection. A test for the presence of COVID-19 yielded a negative result. The patient had been vaccinated, and the vaccination status was confirmed by appropriate documentation.

**Table 1:** Key admission parameters: vital details on day one PCR: polymerase chain reaction; SOFA: Sequential Organ Failure Assessment

Initial laboratory parameters	
COVID-19 PCR	Negative
Blood culture	Negative
Hemoglobin	15,5 g/dl
Leukocyte count	14,100/ul
Lymphocyte count	1,900/ul
Neutrophil count	10,670/ul
Platelets count	253,000/ul
Procalcitonin	0.09 ng/ml
CRP	16.1 mg/l
pH	7.15
HCO_3_	31.4 mmol/l
pCO_2_	132 mm Hg
SatHb	75%
SOFA scale (in points)	6

In the patient's medical history, there was a moderate-severity, multi-week course of COVID-19 infection approximately five months prior. Physical examination revealed evidence of lower extremity post-thrombotic syndrome.

**Figure 1:**
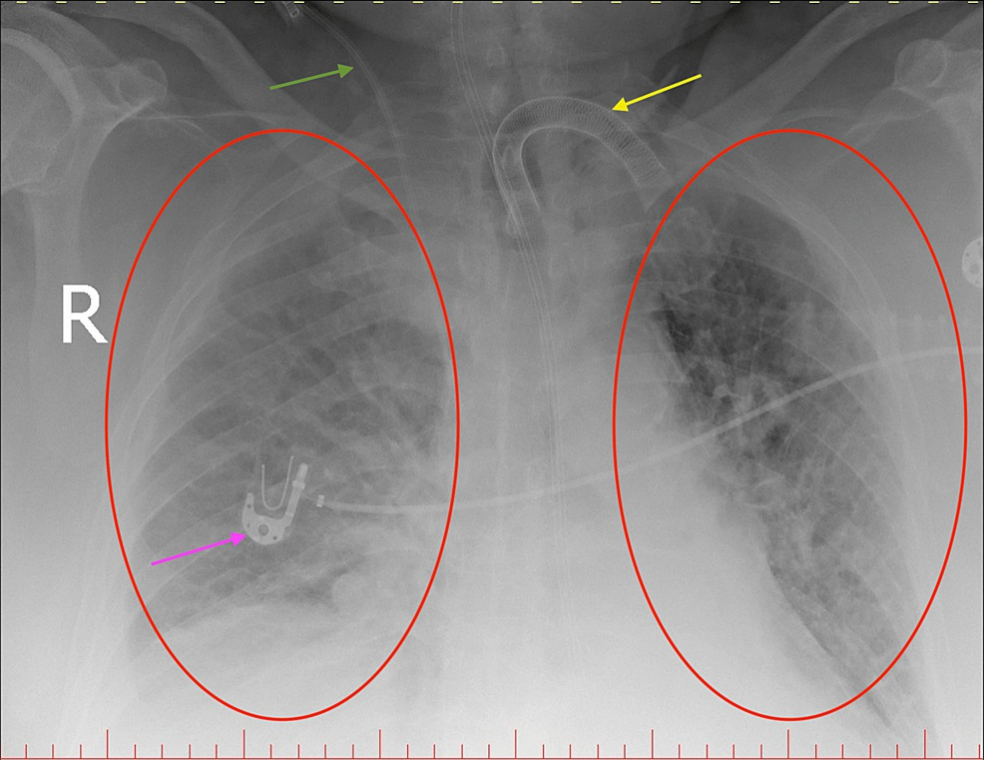
X-ray scan of the chest Red circles: inflammatory changes in both lungs; Pink arrow: ECG electrode; Green arrow: central venous catheter; Yellow arrow: tracheostomy tube

**Figure 2:**
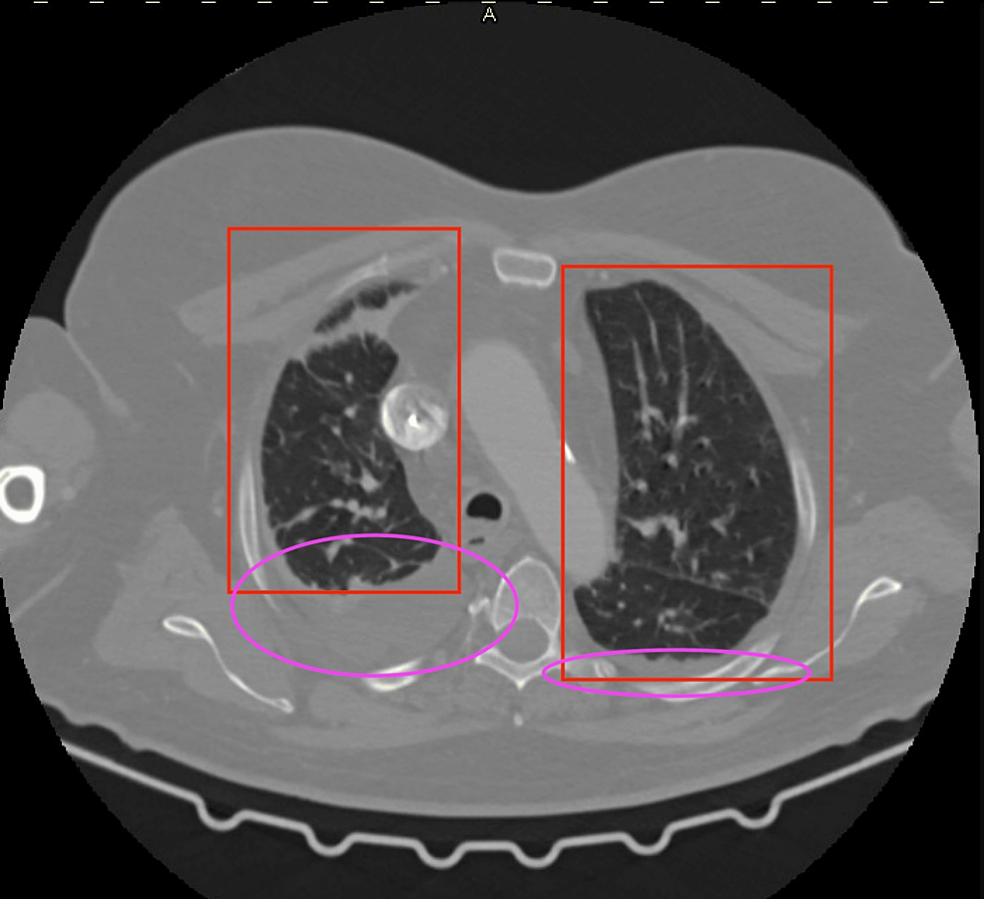
CT scan of the lungs. Upper pulmonary lobes Pink circles: presence of fluid; Red rectangles: typical inflammatory image of lungs after COVID-19 infection

**Figure 3:**
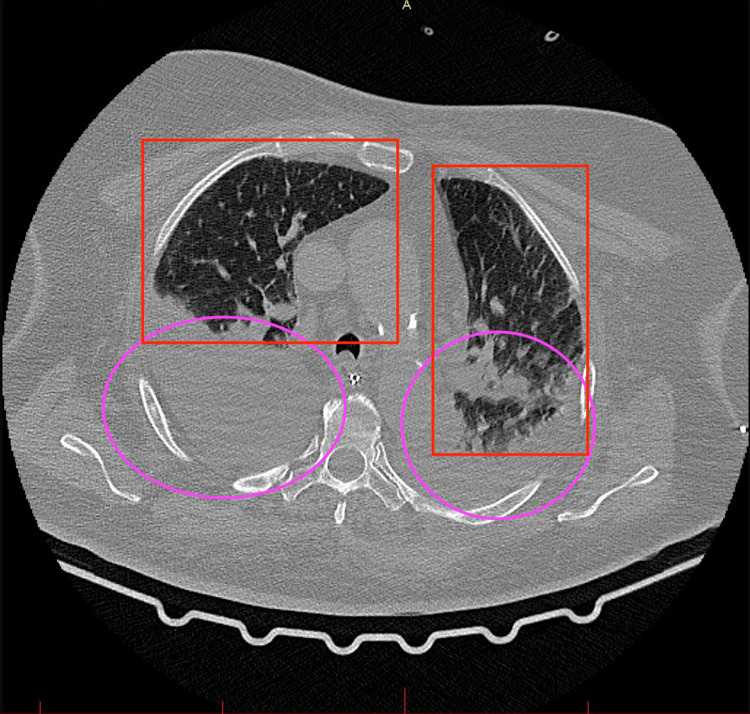
CT scan of the lungs. Lower pulmonary lobes Pink circles: presence of fluid; Red rectangles: typical inflammatory image of lungs after COVID-19 infection

The chest X-ray and computed tomography (CT) scans revealed inflammatory changes in both lungs with the presence of pleural effusion in both pleural cavities (Figures 1-3). No evidence of pulmonary embolism in major and minor pulmonary vessels was observed. Additionally, characteristic findings indicative of pulmonary involvement in the course of COVID-19 infection were detected in ultrasonography (Video 1). The head CT imaging did not reveal any abnormalities.

**Video 1:** Ultrasonography image of pulmonary lesions after COVID-19 infection

Transthoracic echocardiogram (TEE) was performed. TEE conducted three days after admission showed the presence of vegetation on the mitral valve (Videos 2, 3). Anticoagulation therapy with low-molecular-weight heparin was initiated. A week after the first echocardiography investigation, the transthoracic echocardiogram (TTE) examination revealed the absence of vegetation on the valve. The follow-up TEE confirmed the findings observed in the TTE.

**Video 2:** Echocardiography image of vegetation presence on mitral valve

**Video 3:** Echocardiography image of vegetation presence on mitral valve

After ruling out bacterial causes of valvular vegetation, investigations were undertaken to explore alternative etiologies. The possibility of malignancy was eliminated through assessments involving tumor markers, CT imaging, and numerous ultrasound examinations. Laboratory tests utilizing the indirect immunofluorescence (IIF) method indicated the absence of antinuclear antibodies (ANA) and antimitochondrial antibodies (AMA). Systemic lupus erythematosus (SLE) was also ruled out. ELISA testing ruled out the presence of anticardiolipin antibodies in both IgG and IgM titers. On the day of admission, blood cultures were obtained, and bacterial infection was excluded. Considering the echocardiographic findings, the patient's overall condition, and laboratory results, a diagnosis of advanced-stage non-bacterial endocarditis was established.

Given the significant risk of pulmonary embolism (Wells scored 9 points, Geneva scored 13 points) and the detection of vegetation on the mitral valve in the echocardiographic examination, anticoagulant therapy with low-molecular-weight heparin (80 mg/day) was initiated and maintained until the completion of treatment. Echocardiography conducted two weeks after the initiation of anticoagulation therapy revealed normal valves with no evidence of vegetation.

Due to respiratory failure, the patient remained on invasive mechanical ventilation for the entire 85-day hospital stay. Despite multiple attempts, weaning the patient from the ventilator proved unsuccessful.

Throughout the hospitalization, the patient experienced hemodynamic instability, with numerous episodes of hypotension and atrial fibrillation. Due to anuria and signs of renal injury, hemodiafiltration was required.

Upon admission, there were no clinical signs of infection, and both blood cultures and cultures from the upper respiratory tract showed no pathogen growth. However, after 15 days of hospitalization, the patient developed ventilator-associated pneumonia (VAP) caused by* Acinetobacter baumannii *(MDR strain). Despite targeted antibiotic therapy, eliminating the pathogen proved challenging.

Throughout the treatment period, the patient received enteral nutrition, with a total of 1800 kcal/24h administered via continuous infusion through a feeding tube.

Despite an extended 85-day period of intensive care, the implementation of renal replacement therapy, anticoagulation treatment, mechanical ventilation, and nutritional support, the patient's condition progressed toward treatment-resistant multi-organ failure, ultimately leading to the patient's demise.

## Discussion

NBTE is a rare, but clinically significant condition characterized by the presence of sterile thrombi vegetation on heart valves, as confirmed by negative bacteriology examination and the absence of bacterial growth in blood cultures. This medical condition is most commonly associated with malignancy and systemic connective tissue disorders [2].

If left untreated, NBTE can lead to complications such as valvular dysfunction, heart failure, stroke, and systemic embolization [3,9]. The most common embolic complication occurs in the nervous system [3-5]. The pathogenesis involves a hypercoagulable state and the formation of fibrin-platelet thrombi on the altered valve, leading to valve fibrosis, distortion, and subsequent dysfunction [9,10].

Notably, it has been suggested that COVID-19 infection can induce hypercoagulation, potentially contributing to pulmonary embolism, deep venous thrombosis, and the development of NBTE [7,8,11-13].

While a previous COVID-19 infection is certainly one possibility, we do not exclude the possibility that this patient represented one of the cases where isolated NBTE occurred. At the moment, the only institution that has presented a clinical series about patients with cancer-negative and LA/aPLa-negative is the Mayo Clinic [14]. In this specific NBTE group of patients, both mitral and aortic valves are affected. As in the Ca-NBTE group [3], the majority of patients are women [14].

Individuals diagnosed with NBTE should receive long-term anticoagulant therapy to mitigate the elevated risk of systemic embolism and recurrent thromboembolism [5]. In the case of isolated NBTE, the optimal treatment remains unknown, the most common strategy is lifelong warfarin therapy [14].

The objective of this case presentation is to elucidate a distinct cohort of individuals experiencing symptoms of NBTE either during or post-COVID-19 infection, despite the absence of traditionally acknowledged predisposing factors associated with this pathology.

## Conclusions

Non-bacterial endocarditis can manifest independently of malignancy or systemic connective tissue disease. Low molecular weight heparins have shown effectiveness in treating non-bacterial valve vegetations. The term "marantic endocarditis" accurately mirrors the prognosis associated with nonbacterial endocarditis. Moreover, a COVID-19 infection history could be deemed a potential contributing factor to NBTE. Our case contributes to the increasing body of research pinpointing a subgroup of NBTE patients with specific traits, notably those linked to current or previous COVID-19 infection. Given the rarity of these cases, each report offers valuable insights that may help broaden our understanding of the symptoms and characteristics of this disease in the future.
